# Supplementary material for: Simultaneous costs minimizing in electricity and gas micro-grids with the presence of distributed generation
Source: PLoS One. 2024 Sep 19;19(9):e0309999. doi: 10.1371/journal.pone.0309999 (PMC11412674; doi:10.1371/journal.pone.0309999)
Supplement: S1 Appendix — (DOCX) [file pone.0309999.s004.docx]

**Appendix:**

| $\frac{\Pr_{0}}{\xi_{0}Te_{0}}=\frac{\Pr}{\xi Te}=Q^{Gas}Y^{Gas}=Cons\tan t$ | (A-1) |
| --- | --- |
| $\Pr_{ij}^{Aver}.\upsilon_{ij}=\Pr_{0}^{Aver}.\upsilon_{0}\text{ },\text{ }\upsilon_{ij}=\pi d_{ij}^{2}le_{ij}/4$ | (A-2) |
| $\Theta_{ij}^{Initial}=ϒ.\Pr_{ij}^{Aver}.\upsilon_{ij}/\xi_{0}Te_{0}Y^{Gas}.Q^{Gas}\text{ },\text{ }\Theta_{ij,t+1}=\Theta_{ij,t}+\Delta P_{ij,t}^{Gas}.\Delta t$ | (A-3) |
| $\Delta P_{ij,t}^{Gas}=P_{Gas,ijt}^{Gen}-P_{Gas,ijt}^{De}-P_{Gas,ijt}^{Comp}$ | (A-4) |
| $\begin{matrix} f_{ij}^{Gas}=Sgn_{ij}.\xi.\frac{Te_{0}}{\Pr_{0}}.\sqrt{Sgn_{ij}.\frac{\left(P_{i}^{2}-P_{j}^{2}\right).d_{ij}^{5}}{\chi_{ij}.Gr.le_{ij}.Te.Y^{Gas}}}= \\ \text{ }=Sgn_{ij}.W_{ij}.\sqrt{\left(P_{i}^{2}-P_{j}^{2}\right)} \end{matrix}$ | (A-5) |
| $\begin{matrix} Sgn_{ij}=\left\{\begin{matrix} +1\text{ }if\text{ }P_{i}-P_{j}>0 \\ -1\text{ }if\text{ }P_{i}-P_{j}<0 \end{matrix}\right. \\ \zeta=\sqrt{\pi^{2}.\psi^{Air}/64},Gr=\frac{\psi^{Air}}{\psi^{Gas}}=\frac{1}{0.6}=1.6667\text{ },\text{ }\chi_{ij}=\frac{0.032}{d_{ij}^{\left(1/3\right)}} \end{matrix}$ | (A-6) |
| $f_{Gas,ij}^{Copm}=Sgn\left(P_{i},P_{j}\right).\frac{HP_{t,ij}^{Comp}}{l_{1,ij}-l_{2,ij}.\left[\frac{\max\left(P_{i},P_{j}\right)}{\min\left(P_{i},P_{j}\right)}\right]^{l_{3,ij}}}$ | (A-7) |
| $P_{Gas,ijt}^{Comp}\left(HP_{ijt}^{Comp}\right)=\alpha_{1,ij}^{Comp}+\alpha_{2,ij}^{Comp}.HP_{ijt}^{Comp}+\alpha_{3,ij}^{Comp}.\left(HP_{ijt}^{Comp}\right)^{2}$ | (A-8) |
| $PR_{ijt}^{Comp,down}\leq\frac{\max\left(P_{i},P_{j}\right)}{\min\left(P_{i},P_{j}\right)}\leq PR_{ijt}^{Comp,up}$ | (A-9) |
|  | (A-10) |
|  | (A-11) |
|  | (A-12) |
| $Cost_{j,t}^{IL}=\alpha_{1j}^{IL}.\left(P_{jt}^{IL}\right)^{2}+\alpha_{2j}^{IL}.P_{jt}^{IL}$ | (A-13) |
| $Cost_{iM,t}^{BESS}=\left(BLD_{iM}^{BESS}.P_{iM,t}^{BESS}.\Delta t\right)+\left(BLD_{iM}^{BESS}.Es_{iM,t}^{BESS}.\eta^{LIf}\Delta t\right)$ | (A-14) |
| $BLD_{iM}^{BESS}=\frac{BIC}{E_{RC}^{BESS}.LCN^{BESS}}$ | (A-15) |
| $Cost_{iM,t}^{DG}=Co_{iM,t}^{DG}X_{iM,t}^{DG}+SUC_{iM,t}^{DG}X_{iM,t}^{SD}+SDC_{iM,t}^{DG}X_{iM,t}^{SD}$ | (A-16) |
| $Co_{iM,t}^{DG}=\alpha_{1j}^{DG}.\left(P_{iM,t}^{DG}\right)^{2}+\alpha_{2j}^{DG}.P_{iM,t}^{DG}+\alpha_{3j}^{DG}$ | (A-17) |
| $F=\sum_{iM=1}^{M} OBJ_{iM}^{UP}$ | (A-18) |
|  | (A-19) |
|  | (A-20) |
| $\begin{matrix} \sum_{iM=1}^{M} \left(P_{iM,t}^{Wind}+P_{iM,t}^{PV}+P_{iM,t}^{DG}+P_{iM,t}^{BESS}\right)= \\ =\sum_{j=1}^{L} P_{jt}^{De}-\sum_{j=1}^{L} P_{jt}^{IL}+\sum_{iM=1}^{M} P_{iM,t}^{Bid}+P_{t}^{Loss}\text{ }\forall\text{ }t1:T \end{matrix}$ | (A-21) |
| $0\leq P_{iM,t}^{Wind}\leq P_{iM,t}^{Wind,UP}\text{ }\forall\text{ }iM\in1:M\text{ , }\forall\text{ }t\in1:T$ | (A-22) |
| $\frac{P_{iM,t}^{Wind}}{\sqrt{\left(P_{iM,t}^{Wind}\right)^{2}+\left(Q_{iM,t}^{Wind}\right)^{2}}}=Cons\tan t$ | (A-23) |
| $P_{iM,t}^{Wind}\leq P_{iM,t}^{Wind,UP}-\left(\sqrt{\frac{\omega}{1-\omega}}\right)\times\left(\sigma_{P_{iM,t}^{Wind,UP}}\right)$ | (A-24) |
| $P_{iM,t}^{PV}\leq SOL_{t}.S_{PV}.\eta_{PV}\text{ }\forall\text{ }iM\in1:M,\text{ }\forall\text{ }t\in1:T$ | (A-25) |
| $P_{iM,t}^{PV}\leq\left(S_{PV}.\eta_{PV}\right).\left(SOL_{t}-\left(\sqrt{\frac{\vartheta}{1-\vartheta}}\right)\times\left(\sigma_{SOL_{t}}\right)\right)$ | (A-26) |
| $X_{iM,t}^{DG}.P_{iM,t}^{DG}\leq\sum_{l=1}^{NL} P_{iM,t,l}^{DG}\leq X_{iM,t}^{DG}.P_{iM,t}^{DG}\text{ }\forall\text{ }iM\in1:M\text{ },\text{ }\forall\text{ }t\in1:T$ | (A-27) |
| $P_{iM,t}^{DG}\leq P_{Gas,iM,t}^{De,DG}.HRa^{Gas}.\Delta t$ | (A-28) |
| $\sum_{l=1}^{NL} P_{iM,t,1}^{DG}-\sum_{l=1}^{NL} P_{iM,t-1,1}^{DG}\leq Ra_{iM}^{DG,UP}\text{ }if\text{ }\sum_{l=1}^{NL} P_{iM,t,1}^{DG}\geq\sum_{l=1}^{NL} P_{iM,t-1,1}^{DG}$ | (A-29) |
| $\sum_{l=1}^{NL} P_{iM,t-1,1}^{DG}-\sum_{l=1}^{NL} P_{iM,t,1}^{DG}\leq Ra_{iM}^{DG,Down}\text{ }if\text{ }\sum_{l=1}^{NL} P_{iM,t-1,1}^{DG}\geq\sum_{l=1}^{NL} P_{iM,t,1}^{DG}$ | (A-30) |
| $X_{iM,t}^{DG}-X_{iM,t-1}^{DG}\leq X_{iM,t+TU\left(iM,e\right)}^{DG}$ | (A-31) |
| $X_{iM,t-1}^{DG}-X_{iM,t}^{DG}+X_{iM,t+TD\left(iM,e\right)}^{DG}\leq1$ | (A-32) |
| $TU\left(iM,e\right)=\left\{\begin{matrix} e\text{ }e\leq MUT_{iM} \\ 0\text{ }e>MUT_{iM} \end{matrix}\right.\text{ }\forall\text{ }iM$ | (A-33) |
| $TD\left(iM,e\right)=\left\{\begin{matrix} e\text{ }e\leq MUT_{iM} \\ 0\text{ }e>MUT_{iM} \end{matrix}\right.\text{ }\forall\text{ }iM$ | (A-34) |
| $X_{iM,t}^{DG}-X_{iM,t-1}^{DG}\leq X_{iM,t}^{SU}$ | (A-35) |
| $X_{iM,t-1}^{DG}-X_{iM,t}^{DG}\leq X_{iM,t}^{SU}$ | (A-36) |
| $X_{iM,t}^{DG}-X_{iM,t-1}^{DG}\leq X_{iM,t}^{SU}-X_{iM,t}^{SD}$ | (A-37) |
| $\begin{matrix} Es_{iM,t+1}^{BESS}=Es_{iM,t}^{BESS}-P_{iM,t}^{BESS}.\Delta\text{t-}\left\vert P_{iM,t}^{BESS}\right\vert.\eta^{Clf}.\Delta\text{t}- \\ Es_{iM,t}^{BESS}.\eta^{Llf}.\Delta\text{t }\forall\text{ }iM\in1:M\text{ },\text{ }\forall\text{ }t\in1:T \end{matrix}$ | (A-38) |
| $SOC_{iM,t}=\frac{Es_{iM,t}^{BESS}}{E_{RC}^{BESS}}$ | (A-39) |
| $SOC_{iM,t}^{Down}\leq SOC_{iM,t}\leq SOC_{iM,t}^{Up}$ | (A-40) |
| $-\overline{P}_{iM,t}^{Chr,BESS}\leq P_{iM,t}^{BESS}\leq\overline{P}_{iM,t}^{Dis,BESS}$ | (A-41) |
| $Es_{iM,0}^{BESS}=Es_{iM,Initial}^{BESS}\text{ };\text{ }Es_{iM,T}^{BESS}=Es_{iM,End}^{BESS}$ | (A-42) |
| $P_{jt}^{IL}\leq\overline{P}_{jt}^{IL}\text{ }\forall\text{ }j\in1:L\text{ , }\forall\text{ }t\in1:T$ | (A-43) |
| $P_{jt}^{De}\leq P_{jt}^{De}-P_{jt}^{IL}\leq\overline{P}_{jt}^{De}$ | (A-44) |
| $P_{it}^{Inject}\left(V_{it},\theta_{it}\right)+P_{it}^{De}-P_{it}^{IL}=P_{it}^{Elec,Gen}\text{ }\forall t,\text{ }\forall i$ | (A-45) |
| $Q_{it}^{Inject}\left(V_{it},\theta_{it}\right)+Q_{it}^{De}=Q_{it}^{Elec,Gen}\text{ }\forall t,\text{ }\forall i$ | (A-46) |
| $P_{it}^{Inject}=\sum_{j=1}^{L} V_{it}V_{jt}Y_{ij}.\cos\left(\delta_{ij}+\theta_{jt}-\theta_{it}\right)\text{ }\forall t,\text{ }\forall i$ | (A-47) |
| $Q_{it}^{Inject}=-\sum_{j=1}^{L} V_{it}V_{jt}Y_{jt}.\sin\left(\delta_{ij}+\theta_{jt}-\theta_{it}\right)\text{ }\forall t,\text{ }\forall i$ | (A-48) |
| $S_{ij}^{Lower}\leq S_{ijt}\left(V_{it},\theta_{it}\right)\leq S_{ij}^{Upper}\text{ }\forall t,\text{ }\forall i,\text{ }\forall j$ | (A-49) |
| $V_{i}^{\min}\leq V_{it}\leq V_{i}^{\max}\text{ }\forall t,\text{ }\forall i$ | (A-50) |
| $\theta_{i}^{\min}\leq\theta_{it}\leq\theta_{i}^{\max}\text{ }\forall t,\text{ }\forall i$ | (A-51) |
| $P_{Gas,ijt}^{Gen}+\sum_{j=1}^{L} f_{ji}^{Gas}\text{=}\sum_{j=1}^{L} f_{ji}^{Gas}+P_{Gas,ijt}^{De}+P_{Gas,ijt}^{Comp}\text{ }\forall i\in1:L$ | (A-52) |
| $P_{Gas,ijt}^{De}=P_{Gas,iM,t}^{De,DG}+P_{Gas,it}^{De,Heat}$ | (A-53) |
| $\underline{\Theta}_{ij,t}\leq\Theta_{ij,t}\leq\overline{\Theta}_{ij,t}$ | (A-54) |
| $\Theta_{ij,0}=\Theta_{ij,Initial};\Theta_{ij,T}=\Theta_{ij,End}$ | (A-55) |
| $\underline{P}^{Exchange}\leq P_{iM,t}^{Bid}\leq\overline{P}^{Exchange}\text{ }\forall t\in1:T$ | (A-56) |
| $\Delta P_{t}^{\mathrm{Im}ba}=P_{t}^{RT}-\sum_{iM=1}^{M} P_{iM,t}^{Bid}$ | (A-57) |
| $P_{t}^{RT}=\sum_{iM=1}^{M} \left(P_{iM,t}^{Wind}+P_{iM,t}^{PV}+P_{iM,t}^{DG}+P_{iM,t}^{BESS}\right)+\sum_{j=1}^{L} P_{jt}^{IL}-\sum_{j=1}^{L} P_{jt}^{De}$ | (A-58) |
| $Cost_{t}^{\mathrm{Im}ba}=\left\{\begin{matrix} \rho_{t}^{\mathrm{Re}+}.\Delta P_{t}^{\mathrm{Im}ba}\text{ },\text{ }\Delta P_{t}^{\mathrm{Im}ba}<0 \\ \rho_{t}^{\mathrm{Re}-}.\Delta P_{t}^{\mathrm{Im}ba}\text{ },\text{ }\Delta P_{t}^{\mathrm{Im}ba}\geq0 \end{matrix}\right.$ | (A-59) |
