# Supplementary material for: Simultaneous costs minimizing in electricity and gas micro-grids with the presence of distributed generation
Source: PLoS One. 2024 Sep 19;19(9):e0309999. doi: 10.1371/journal.pone.0309999 (PMC11412674; doi:10.1371/journal.pone.0309999)
Supplement: Attachment — Submitted filename: Response to reviewers_1.doc [file pone.0309999.s005.doc]

Dear Editor,

Thank you so much for sending the reviewers comments related to manuscript entitled “Simultaneous Costs Minimizing in Electricity and Gas Micro-grids with the Presence of Distributed Generation”. We applied the reviewers’ comments to improve the quality of the paper. Responses to the reviewers’ comments are presented in the same order as the comments have been addressed.

Reviewer #1:

Thank you so much for your time and valuable comments. Based on your comments, the paper has been revised and edited. The revisions made according to your comments are as followed:

1) The abstract is very long, write this section briefly.

**Answer:**

According to this comment, the abstract has been shortened as much as possible.

2) Compare your work with other works in a table in the article.

**Answer:**

According to this comment, the comparison between this paper and other works has been added as follows:

**Added text (section introduction):**

By reviewing the references and stating the goals of each one, the summary of the work done and its comparison with the innovations of this paper is shown in Table (1). According to this table, it is clear that the simultaneous examination of gas and electricity in the network, considering different types of load to analyze the results, and considering the energy hub management are among the innovations of this article that are less discussed in previous works. Also, the type of objective function in this work is one of its other specific innovations.

Table (1): Compare the proposed method and previous methods

| **No.** | **Aspect** | **[19]** | **[20]** | **[21]** | **[22]** | **[23]** | **[24]** | **[25]** | **[29]** | **[30]** | **[31]** | **This paper** |
| --- | --- | --- | --- | --- | --- | --- | --- | --- | --- | --- | --- | --- |
| **1** | **Energy Losses** |  | **✓** | **✓** |  | **✓** |  |  | **✓** | **✓** |  |  |
| **2** | **Prediction Model** |  |  |  |  | **✓** | **✓** | **✓** |  |  |  |  |
| **3** | **EV Considering** |  |  |  |  |  |  |  |  |  | **✓** |  |
| **4** | **Unbalance Load** | **✓** | **✓** | **✓** |  | **✓** |  |  |  |  |  |  |
| **5** | **Demand Response Considering** |  |  |  |  |  |  |  | **✓** | **✓** | **✓** |  |
| **6** | **Feeder Reconfiguration** |  |  |  |  |  |  |  | **✓** | **✓** |  |  |
| **7** | **Voltage Stability Considering** |  |  |  |  |  |  |  | **✓** | **✓** |  |  |
| **8** | **ES & different DGs Considering** |  | **✓** | **✓** | **✓** | **✓** | **✓** | **✓** | **✓** | **✓** | **✓** | **✓** |
| **9** | **Multi-Objective Analysis** | **✓** | **✓** | **✓** | **✓** | **✓** |  |  | **✓** | **✓** | **✓** | **✓** |
| **10** | **24-Hours Analysis** | **✓** | **✓** | **✓** | **✓** | **✓** |  |  | **✓** | **✓** | **✓** | **✓** |
| **11** | **EH Managing** |  |  |  |  |  |  |  |  |  | **✓** | **✓** |
| **12** | **Load Types Considering** |  |  |  |  |  |  |  |  |  |  | **✓** |
| **13** | **Simultaneous gas & Electricity Considering** |  |  |  |  |  |  |  |  |  | **✓** | **✓** |
| – EV: Electrical Vehicle – ES: Energy Storage – DG: Distributed Generation – EH: Energy Hub | | | | | | | | | | | | |

3) It is suggested to check the following studies in the introduction:

\# <https://doi.org/10.1002/er.5709>

#https://doi.org/10.1007/s12652-020-02322-2

#https://doi.org/10.1155/2023/9042957

**Answer:**

Thanks for introducing suitable articles in this field. These articles have been studied in full detail and mentioned in the article. Also, a comparison has been made between these articles and the present article which has been mentioned as follows:

**Added text (section introduction):**

In distribution networks, the voltage stability index is an important issue and is considered as a security objective function. Therefore, it is necessary to check this index by energy management in the distribution network. According to [29], this index is taken into account and dynamic distribution feeder reconfiguration (DDFR) is introduced as a suitable method for this management. This study has been considered by considering the voltage stability index, power loss and operation cost in a distribution network with the presence of PV, energy storage system and capacitors.

In distribution networks, electricity prices and load patterns are constantly changing, and accordingly, many operational problems will affect electricity distribution networks. Therefore, in order to prevent these incidents, the problem will be considered in different time frames. In [30], a multi-objective optimization model is presented using the DDFR procedure in the distribution system and in multiple time intervals with the presence of energy storage systems and PV units. In [31], a cost-effective energy hub system is proposed using linear programming, considering transformer converters, combined heat and power (CHP), heat exchangers, and energy storage, including electric vehicle charging stations (EVCSs), and heat storage.

[29] Lotfi H. Multi-objective energy management approach in distribution grid integrated with energy storage units considering the demand response program. International Journal of Energy Research, Vol. 44, No. 13, 2020.

[30] Lotfi H, Ghazi R. Optimal participation of demand response aggregators in reconfigurable distribution system considering photovoltaic and storage units. Journal of Ambient Intelligence and Humanized Computing, Vol. 12, pp. 2233–2255, 2020.

[31] Nikkhah MH, Lotfi H, Samadi M, Hajiabadi ME. Energy Hub Management considering Demand Response, Distributed Generation, and Electric Vehicle Charging Station. Vol. 2023, 2023.

4) What is the main difference between your work and other works? In other words, specify the innovation of the article clearly in the article.

**Answer:**

According to this comment, the innovations of the article are fully and clearly stated in the introduction section and highlighted in Table (1).

5) The language of the article needs to be revised.

**Answer:**

According to this comment, the language of manuscript has been revised, completely. Corrections are highlighted.

6) Analyzing the results is not enough. In other words, the explanation section about the results is incomplete.

**Answer:**

According to this comment, more analysis of the results is done in this section and highlighted.

7) What test network is used in the article?

**Answer:**

According to this comment, additional explanation of test network has been added to the article as follows:

**Added text (section 4):**

The simulation performed in this paper was tested on a 33-bus IEEE radial distribution system and its information can be accessed in [32]. The general schematic of this micro-grid is according to Fig. (2). Also, the load characteristics of this network have been used based on [33].

8) The data related to solar and wind were extracted from which study?

**Answer:**

According to this comment, additional explanations of wind and solar data have been added to the article as follows:

**Added text (section 4):**

The safety pressure and diameter for gas pipelines are 140 kPa and 660 mm, respectively. Information related to wind turbine sources as well as distributed generation units is given in [34]. In addition, the price of natural gas can be seen in [35].

9) The conclusion does not support the simulation results.

**Answer:**

According to this comment, conclusion has been revised as follows:

**Edited text (section conclusion):**

In this article, an attempt was made to code the simulation in the form of an MILP using GAMS software. Based on this and according to the output of this software, the amount of power difference between generators and load using GAMS problem optimization, the charge and discharge values of energy storage have been expressed. In other words, this difference is the lack or excess of power in the network, which needs to be charged and discharged by the energy storage source. The results of this optimization were verified according to the charging and discharging output of the energy storage device. The relative difference was caused by the inertia of the energy storage source. By precisely defining the optimization problem and extracting the objective function, the amount of production of each electrical energy generating unit with two issues of electricity and gas during 24-hours was accurately obtained. According to the results of this article, it is possible to accurately determine the amount of production of each resource to reach the optimal cost amount during the 24-hours. With this analysis, network costs will be significantly reduced by combining gas and electricity. In other words, it is possible to go to gas power plants during the hours when electricity is needed and use relatively cheaper energy such as solar energy during the day, thus increasing network costs. With optimal energy consumption and management of the production side, network costs can be optimized and this cost can be transferred to investment in other sectors. The method proposed in this article is based on mathematics and due to the use of software based on optimization, the accuracy of its results is high.

Reviewer #2:

Thank you so much for your time and valuable comments. Based on your comments, the paper has been revised and edited. The revisions made according to your comments are as followed:

1) The manuscript needs substantial revision for language and grammar.

"scattered production"= Distributed generation

"This article is done on a multi-objective network"

"transformation of passive and passive"

"these two energies" these two energy carriers"

"mixed correct nonlinear programming" = mixed integer linear programming known as MILP.

"Gems"=GAMS

**Answer:**

According to this comment, the manuscript has been revised, completely. Corrections are highlighted.

2) Using appendix for equations makes the review of the paper hard.

**Answer:**

According to the large number of mathematical equations and also the perception of these equations from previous references (and not the innovative equations of the paper), it has been tried to place these equations in the appendix so that the paper focuses on the main proposed and innovative topic.

3) Overall the paper is not well organized.

**Answer:**

According to this comment, the structure and writing of the paper has been revised to an acceptable extent. Corrections are highlighted.
