# Supplementary material for: Simultaneous costs minimizing in electricity and gas micro-grids with the presence of distributed generation
Source: PLoS One. 2024 Sep 19;19(9):e0309999. doi: 10.1371/journal.pone.0309999 (PMC11412674; doi:10.1371/journal.pone.0309999)
Supplement: Attachment — Submitted filename: Response to Editor.doc [file pone.0309999.s006.doc]

Dear Editor,

Thank you so much for sending the reviewers comments related to manuscript entitled “Simultaneous Costs Minimizing in Electricity and Gas Micro-grids with the Presence of Distributed Generation”. We applied the reviewers’ comments to improve the quality of the paper.

- Based on this, the grammatical points and writing errors of the article have been corrected in general.
- Also, the relevant references have been explained more carefully and modified in the article.

Responses to the reviewers’ comments are presented in the same order as the comments have been addressed.

Reviewer #1:

Thank you so much for your time and valuable comments.

Reviewer #2:

Unfortunately, your comments were not sent to the authors so that the comments are applied and the paper is corrected. However, according to the editor's opinion, the points were revised and completely revised.

Reviewer #3:

Thank you so much for your time and valuable comments.
